# Preliminary Study on the Effect of a Single High-Energy Electromagnetic Pulse on Morphology and Free Radical Generation in Human Mesenchymal Stem Cells

**DOI:** 10.3390/ijms24087246

**Published:** 2023-04-14

**Authors:** Joanna Czwartos, Bernadeta Dobosz, Wiktoria Kasprzycka, Paulina Natalia Osuchowska, Małgorzata Stępińska, Elżbieta Anna Trafny, Jacek Starzyński, Zygmunt Mierczyk

**Affiliations:** 1 Institute of Optoelectronics, Military University of Technology, 2 Kaliskiego St., 00-908 Warsaw, Poland; 2 Faculty of Physics, Adam Mickiewicz University, Uniwersytetu Poznańskiego 2, 61-614 Poznań, Poland; 3 Faculty of Electronical Engineering, Warsaw University of Technology, Koszykowa 75, 00-662 Warsaw, Poland

**Keywords:** electromagnetic pulse (EMP), reactive oxygen species (ROS), human mesenchymal stem cells (hMSC), electron paramagnetic resonance (EPR), scanning electron microscope (SEM), Marx generator

## Abstract

The effect of nanosecond electromagnetic pulses on human health, and especially on forming free radicals in human cells, is the subject of continuous research and ongoing discussion. This work presents a preliminary study on the effect of a single high-energy electromagnetic pulse on morphology, viability, and free radical generation in human mesenchymal stem cells (hMSC). The cells were exposed to a single electromagnetic pulse with an electric field magnitude of ~1 MV/m and a pulse duration of ~120 ns generated from a 600 kV Marx generator. The cell viability and morphology at 2 h and 24 h after exposure were examined using confocal fluorescent microscopy and scanning electron microscopy (SEM), respectively. The number of free radicals was investigated with electron paramagnetic resonance (EPR). The microscopic observations and EPR measurements showed that the exposure to the high-energy electromagnetic pulse influenced neither the number of free radicals generated nor the morphology of hMSC in vitro compared to control samples.

## 1. Introduction

Over the last 20 years, rapid progress in the development of electronic devices and systems, including computers and, above all, telecommunications equipment has been observed. All these devices are widely used in various areas of human activity, ranging from entertainment and commerce, through science, industry, banking, and health care, to state administration and the armed forces. All these devices are sources of electromagnetic radiation, and, as their use in everyday life increases, so does the exposure of the human body to many types of electromagnetic fields (EMF). The influence of electromagnetic fields, i.e., non-ionizing radiation on living organisms, has been the subject of many scientific investigations. However, the results of these studies are still subject to constant discussion and often appear to be contradictory.

The majority of studies presented in the literature mainly concern the health effects of exposure to the fields emitted by cell phones (RF EMF; radiofrequency electromagnetic fields), and the relationship between exposure and the occurrence of neoplasms in the human head (including glioblastoma, meningioma, acoustic neuroma, eye melanoma, etc.). Early studies on this issue suggested, for example, an increased risk of acoustic neuroma [1,2,3]. However, the latest analyses based on large-scale prospective studies showed no convincing evidence that the normal use of cell phones increases the incidence of brain tumors [4,5,6].

Other studies have reported that exposure to micro- and radiofrequency fields may be, in addition to a cancer risk, associated with cardiovascular diseases (such as blood pressure disorders, ventricular arrhythmia, etc.) or nervous system diseases, including neurodegenerative ones such as amyotrophic lateral sclerosis (ALS) [7,8,9], and Huntington’s disease (HD) [10].

One specific type of electromagnetic field is the short, high-energy electromagnetic pulse (EMP), characterized by an extremely fast rising time and a broad bandwidth. This type of pulse can be triggered by a single nuclear explosion (in this case, the generated radiation is ionizing radiation, which is very harmful to human health), lightning strike, or through non-nuclear devices, i.e., various high-power generators or reactive chemicals. Pulses that do not generate nuclear radiation are often used as weapons in cyber warfare or cyberterrorism. They can cause disruption to communication systems, temporary or permanent damage to technical devices (so-called “soft kill” or “hard kill”), or important objects of the critical infrastructure. The studies on pulses used as weapons are usually classified and therefore not accessible. However, in this case, as in the case of the exposure to non-pulsed electromagnetic fields, there is a concern that this type of pulsed field may have adverse health effects. Ding et al. (2009) [11] observed that exposure to 200 and 400 electromagnetic pulses at 200 kV/m increased cerebral micro-vascular permeability in rats. These effects, however, were reversible. On the other hand, Zeng et al. (2011) [12] observed that the EMP irradiation of rats led to a deterioration in their fertility, and, more specifically, to morphological damage to the rats’ testes, as well as endocrine and metabolic disorders. Other studies have also confirmed unfavorable bioeffects, such as a significant alteration in the arterial blood pressure of rats when exposed to 200 and 400 EM pulses at 200 kV/m and 400 kV/m [13], and significant decreases in the associative learning abilities of mice (when exposed to 200 pulses at 400 kV/m) [14].

Many scientific works have reported that EMF/EMP can lead to an excessive increase of free radicals in organisms [15,16,17]. The overproduction of reactive oxygen species (ROS) leads to the destruction of almost all cell components. The components of cell membranes are particularly susceptible to damage—mainly polyunsaturated fatty acid residues, which are destroyed in the lipid peroxidation process induced by ROS. It results in the disintegration of the membranes and the entire contents of the cells “spilling out” and initiating widespread inflammation. Not only biological membranes, but proteins and nucleic acids are damaged as well. Consequently, it may lead to many neurodegenerative diseases (Alzheimer’s disease, Parkinson’s disease) as well as the induction of new, or the acceleration of existing, neoplastic processes [18,19].

The only known direct method that enables the measurement of free radicals is electron paramagnetic resonance (EPR) [20]. This method has been successfully applied in previous studies on free radicals in biological materials, such as blood samples [21,22,23], and in the studies of radical processes occurring in both healthy and cancer cells [24,25,26].

As is known, free radicals are characterized by high reactivity and a very short lifetime [27]. Therefore, in biological specimen studies, such as the cells or blood mentioned above, substances that allow radicals to be “trapped” in the sample—spin traps—are very often used. Which trap is used depends on the type of radicals to be trapped [20]. The resulting adduct is stable, which facilitates its measurement. One of the known spin traps is 1-hydroxy-3-methoxycarbonyl-2,2,5,5-tetramethylpyrrolidine (CMH). It is often used, for example, in studies of free radicals in blood samples [28,29,30,31].

The aim of the study was to examine the effect of a single electromagnetic pulse on the viability, morphology, and reactive oxygen species generation in human mesenchymal stem cells (hMSC). Undifferentiated multipotent primary stem cells isolated from bone marrow are considered a good research model. Mesenchymal cells are present in all multicellular organisms and play an important role in regeneration processes due to their ability to self-renew or differentiate into chondrogenic, osteoblastic, or mesenchymal adipose tissues. Furthermore, hMSC adhere well to plastic in culture vessels, so they could be easily cultured in vitro. hMSC derived from different sources have been well characterized for the isolation procedure, their multilineage differentiation capacity, and their phenotypic characterization [32]. It has been shown that exposure to a pulsed electromagnetic field can affect cell viability, proliferation, and differentiation, altering many metabolic pathways of the stem cells [33].

The viability and morphological studies were carried out using a confocal fluorescence microscope and scanning electron microscope (SEM), respectively, while the examination of the ROS number in the samples was performed using electron paramagnetic resonance (EPR) spectroscopy. In this paper, an innovative configuration of a system for generating a high-energy electromagnetic pulse (based on a Marx generator), which was used for the hMSC treatment, is presented for the first time. The innovation relied on proper tuning (using Ansys Electronic Desktop computer simulations), the Marx generator, and the stripline to obtain the best possible parameters (steepness of rise and amplitude) of the electromagnetic field pulse. All measurements were made for two-time points: 2 h and 24 h after the exposure to EMP in two independent experiments (with at least three biological repetitions in each experiment).

## 2. Results 

### 2.1. The hMSC Viability and Morphology

**Figure 1:**
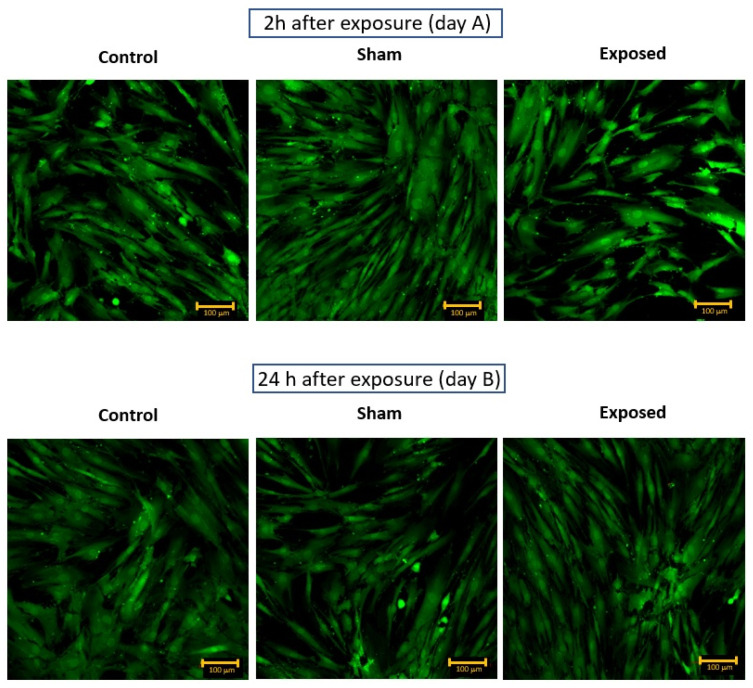
The viability of hMSC. Experiment no. 1: day A—2 h after exposure to EMP and day B—24 h after exposure to EMP.

The mesenchymal cell viability in our study was monitored by a confocal fluorescence microscope using a double-staining assay (LIVE/DEAD™ Viability/Cytotoxicity Kit, Invitrogen™, Waltham, MA, USA). Red nuclei, representing the cells with damaged cell membranes, were not observed in any sample. All the cells were bright green, indicating the high esterase activity. Spindle and large, flattened cells were observed 2 h after the exposure as well as in control and sham samples (Figure 1, day A).

Further monitoring of the hMSC culture after 24 h showed no changes in the metabolic activity or shape of the cells (Figure 1, day B).

**Figure 2:**
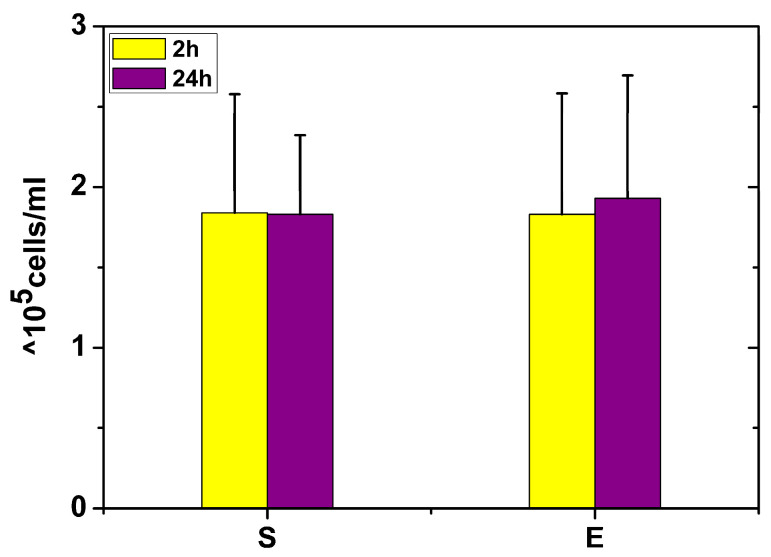
The number of live hMSC in sham and exposure probes after detachment from the culture vessel 2 h and 24 h after exposure to EMP (cells/mL). Measurements were made in two independent experiments with three independent biological specimens exposed in each experiment.

Similar viability results were obtained after measuring the number of live hMSC in sham and exposure probes following detachment from the culture vessel after exposure to EMP (Figure 2).

No significant changes in the quantity of live cells were observed after 24 h compared to the samples observed just 2 h post exposure to a single high-energy electromagnetic pulse.

**Figure 3:**
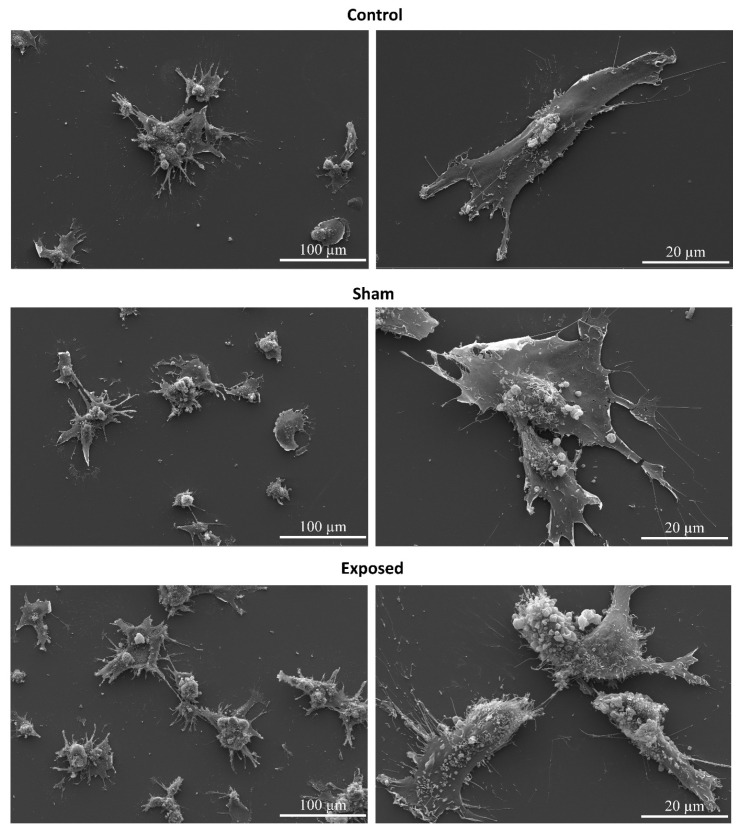
The morphology of hMSC. Experiment no. 1: day A—2 h after exposure to EMP. Scale bars represent the image magnification.

A scanning electron microscope was used to monitor the hMSC morphology (Figure 3). hMSC show different morphological shapes with predominated elongated cells in all samples. Moreover, exposed and unexposed cells had many microvilli on their surface together with numerous protrusions, which is characteristic of healthy mesenchymal cells. 

### 2.2. EPR Studies

**Figure 4:**
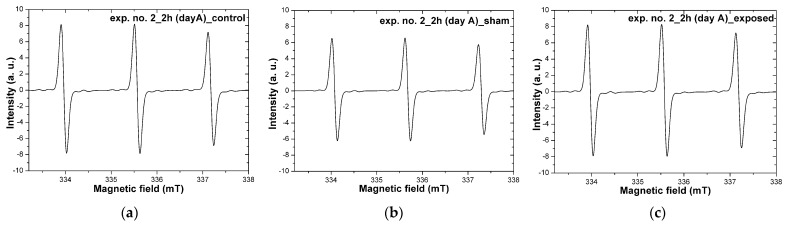
Representative EPR spectra of hMSC with CMH spin trap recorded at 2 °C: control sample (**a**), sham sample (**b**), and exposed sample (**c**).

In this method, the sample is placed in a resonant cavity between the pole pieces of the electromagnet. Under such conditions, the spins of the unpaired electrons and their magnetic moments are oriented in parallel or antiparallel to the direction of the magnetic field. Electromagnetic radiation in the microwave range delivered to the system, corresponding to the energy difference between these orientations and thus meeting the resonance condition (1), is absorbed, and the EPR spectrum is observed as a result of this phenomenon [34]:

(1) $$h\nu =g\mu_{B}B,$$

(where: h is a Planck constant, ν is a microwave frequency, g is a spectroscopic splitting factor characteristic for each free radical, $\mu_{B}$ is a Bohr magneton, and B is the induction of the magnetic field). This method was used to monitor the generation of free radicals in mesenchymal cells after a single electromagnetic pulse exposure by recording the EPR spectra. In turn, to check the reproducibility of the results, independent experiments were performed twice, with three repetitions in each of them (exp. no.1 and no.2). Figure 4 shows representative EPR spectra for the control sample (Figure 4a), sham sample (Figure 4b), and for the exposed sample (Figure 4c). The three lines visible in all three spectra result from the interaction of the unpaired electron with the nitrogen nucleus (Figure in Section 4.4.1), for which the spin equals 1. The characteristic value of the spectroscopic splitting factor (g)—a parameter characterizing a given radical—was equal to 2.0055, and the so-called hyperfine interaction constant (A) —giving information about the interaction of the spin of the unpaired electron with the nucleus spin—was equal to 1.6 mT. These parameters did not change for individual spectra (for the control, sham, and exposed samples).

**Figure 5:**
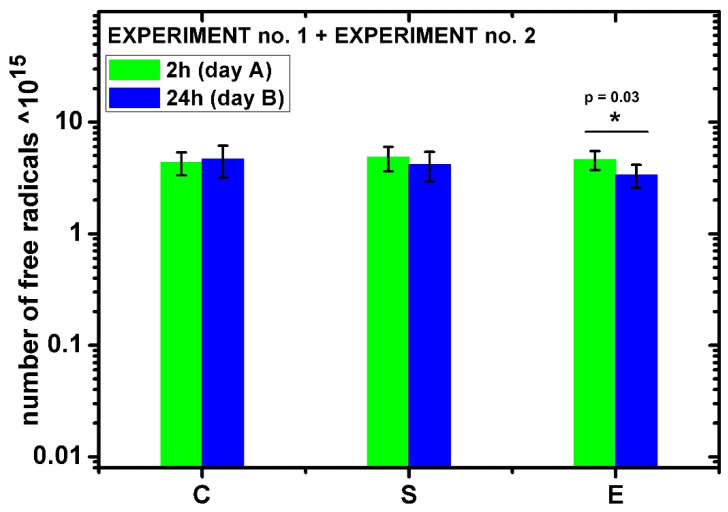
Representative EPR spectra of hMSC with CMH spin trap recorded at 2 °C: control samples (C, *n* = 6), sham samples (S, *n* = 6), and exposed samples (E, *n* = 6); * *p* = 0.03 for exposed samples after 2 h and 24 h. Measurement was performed in two independent experiments (with three independent biological specimens exposed in each experiment).

The EPR spectra were also analyzed quantitatively by determining the number of free radicals in each sample. The summed results obtained from two independent experiments (with at least three biological repetitions in each experiment) are shown in Figure 5. There were no statistically significant differences between the groups of samples: control, sham, and exposed. Only for the samples treated with a high-energy single pulse at 2 h and 24 h after exposure was a statistically significant difference observed at the level of *p* = 0.03 (Student’s *t*-test). For this group, a decrease in the number of free radicals was observed after 24 h, i.e., from 4.6 × 10^15^ ± 0.9 × 10^15^ (2 h after exposure) to 3.3 × 10^15^ ± 0.8 × 10^15^ (24 h after exposure). 

### 2.3. Simulations

**Figure 6:**
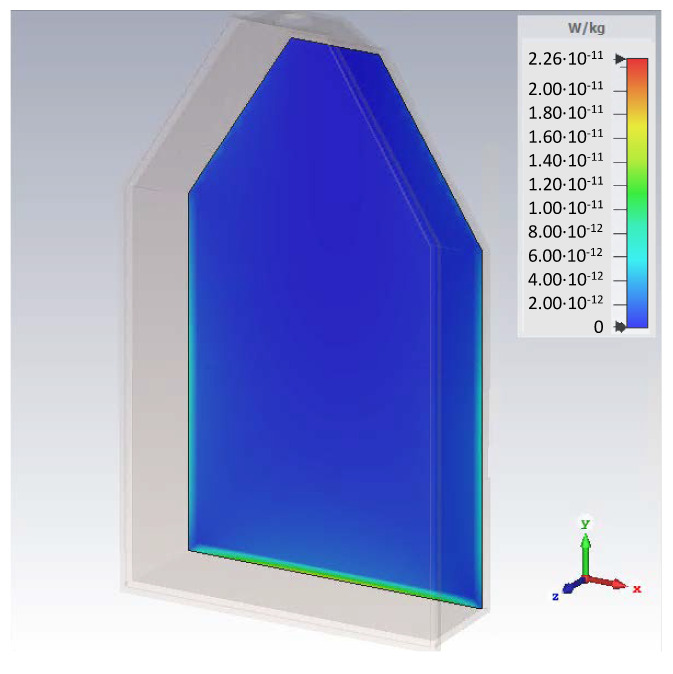
SAR distribution in a thin layer of bone marrow cells grown in the culture flask T175 (spatial visualization).

The specific absorption rate (SAR) distribution in a thin layer of biological material (bone marrow cells) grown in the culture flask T175 was obtained based on a numerical simulation. The maximum SAR values obtained were ~3.5 × 10^−11^ W/kg (Figure 6).

Based on the simulation data, no thermal effect on the tested biological material was found.

## 3. Discussion

### 3.1. The hMSC Viability and Morphology 

No viability or morphology changes were observed between the exposed sample and the controls in experiment no. 1. Healthy green cells with active intracellular esterase and intact membranes were observed in all the exposed samples after staining with fluorescence dyes. Similar results on the viability and morphology of hMSC were also obtained in experiment no. 2. To support this observation, no decrease in the cell metabolic activity was observed. Additionally, a comparable number of viable detached cells measured in samples 2 h and 24 h post exposure indirectly indicated that exposure to a single high-energy electromagnetic pulse in our arrangement did not affect the viability of hMSC. 

Scanning electron microscope observations showed that hMSC were of three cellular shapes: small triangular with raised cytoplasmic regions, elongated spindle-shaped, and large and flattened with a sizeable clearly visible nucleus [35]. Elongated cells predominated in all samples. Since large, flattened cells are considered to have a reduced differentiation potential [35,36], the presence of a predominant fraction of elongated, prone-to-differentiation cells in all our samples (pre- and post-exposure) might indicate that the exposure to a single electromagnetic pulse did not change the differentiation potential of hMSC. However, the limitation of our study is the lack of demonstration of hMSC’s capability to differentiate into adipocytes, osteoblasts, and chondrocytes after exposure to a single high-energy electromagnetic pulse. The rationale behind such experimentation is that oxidative stress may lead to premature senescence or shorten the lifespan of hMSC. Even though we did not observe an increase in free radicals in our experiments, demonstrating the unperturbed differentiation potential would confirm the undisturbed homeostasis of hMSC exposed to a single EMP.

Moreover, the exposed and unexposed cells had many microvilli on their surface, together with numerous protrusions, which is characteristic of healthy mesenchymal cells. It was previously reported that pulsed electromagnetic fields could change the morphology of hMSC [37]. The hMSC volume might increase after the treatment with the EMP (5–150 Hz, 1.1 mT). Their shape might become more triangular and polygonal, and granular materials could be noticed on their surface. However, in our study, no morphological changes were observed for hMSC 2 h after exposure to a single high-energy electromagnetic pulse with a magnitude reaching ~1 MV/m compared to control and sham cells.

### 3.2. EPR Examinations 

The EPR spectra were analyzed quantitatively by determining the number of free radicals in each sample. A statistically significant difference was observed at 2 h and 24 h after exposure only for the group of exposed samples (E) treated with a single high-energy electromagnetic pulse. The cells generated fewer free radicals after 24 h than immediately after exposure. The biological mechanism of this phenomenon has yet to be explained. It requires further research using, for example, techniques such as DNA microarrays to determine the expression profile of all genes in the studied cells. 

In the literature, there are reports of similar studies using electromagnetic pulses and biological materials, but only one study was found in which the EPR method was used [38]. The authors of this study investigated the effect of non-ionizing electromagnetic radiation pulses on free radical production in rat liver mitochondria. Their research showed that EMP was rather protective against generating free radicals. The authors suggested that this might be because EMP reduces oxygen consumption, and it prevents the generation of free radicals. In the authors’ opinion, EMP changes the mobility of membrane proteins, and mitochondrial functions, such as respiratory chain and oxygen consumption, are affected.

On the contrary, Alkis et al. [16] and Kesari et al. [15] observed that radiofrequency radiation caused oxidative stress in rat brains. In their studies, however, the EPR method was not used, only the calorimetric methods, based on which the degree of oxidative stress was calculated [16], and spectrophotometric methods for ROS measurements were used [15]. Another study also investigated the effects of EMP on the central nervous system in rats, and malondialdehyde (MDA) was used as a biomarker of oxidative stress [39]. The authors showed that sevoflurane has a neuroprotective effect against EMP-induced brain damage by inhibiting neuronal oxidative stress.

The research results described so far in the literature suggest that EMP radiation may affect the proliferation and differentiation of cells and the level of free radicals [40]. There are also reports on both the pro-oxidative and neuroprotective effects of electromagnetic fields [41]. Therefore, learning about this mechanism requires further research [40]. So far, studies on the effects of EMP have mainly been carried out on cell lines and animals, so it is suggested that studies involving humans should also be considered to better estimate its risk to human health [42].

Based on the EPR results, this study concluded that the applied single pulse with an electric field magnitude of ~1 MV/m did not affect the generation of free radicals in hMSC compared to control samples. It is consistent with the biological results presented earlier in this paper.

## 4. Materials and Methods

### 4.1. Human Mesenchymal Stem Cell Culture

hMSC were obtained from the bone marrow of a healthy 22-year-old adult Caucasian man (PT-2501 Lonza, Houston, TX, USA). The hMSC suspensions from passage “3” were the starting material for the experiments performed. Cells were recovered from the frozen vial and resuspended in Mesenchymal Stem Cell Growth Medium^TM^ (MSCGM) BulletKit^®^ (PT-3001, Lonza, MD, USA). The adherent cells were cultured in a CO_2_ incubator (INCOmed 153, Memmert, Germany) in an atmosphere of 90% humidity, at a temperature of 37 °C and at 5% CO_2_ up to a confluence of 70–80%. The medium changes were performed every 2–3 days, and the morphology of adherent cells was analyzed using an inverted light microscope (Nikon Ts2R-FL, Nikon Instruments Inc., Basel, Switzerland). The cells were stained with trypan blue and counted using a Countess^®^ Automated Cell Counter (Invitrogen, Carlsbad, CA, USA). hMSC diluted to the appropriate density were plated into 18 T175 cell culture flasks (Nunc^®^, Thermo Fisher Scientific, Waltham, MA, USA) (at a seeding density of 3 × 10^5^ cells per 175 cm^2^), nine for day A (the day of the electromagnetic pulse exposure) and nine for day B (24 h after the electromagnetic pulse exposure) of the experiments. Additionally, culture plates (24-well, Greiner Bio-One Germany) were seeded with 2 × 10^3^ cells/well for microscopic observations. Three types of the samples were examined: exposed, sham, and control samples running concomitantly at two time points of day A and day B of each experiment (2 h and 24 h after EMP exposure). The adherent cells were exposed to a single electromagnetic pulse in T175 culture flasks and 24-well culture plates. After exposure, the adherent cells were immediately washed with a 10 mL PBS buffer without magnesium and calcium ions (D-PBS, Dulbecco’s-PBS, Gibco, NY, USA) and detached from suitable culture vessels by trypsinization with trypsin/ethylenediaminetetraacetic acid (Trypsin/EDTA, CC-3232, Lonza, Walkersville, MD, USA) in a volume of 4 mL per T175 bottle. After the cells were detached, trypsin was inhibited with a culture medium with 2% fetal bovine serum (FBS, LONZA S1810-500, Walkersville, MD, USA) at 10 mL per bottle. The cell pellet was washed with PBS and resuspended in a fresh MSCGM medium. On day A of the experiments, the 10 mL of cell suspension from each flask constituted the suitable tested sample. The number of live detached hMSC from each sample tested was counted after tryptan blue staining with the Countess^®^ Automated Cell Counter. For day B, hMSC were cultured for the next 24 h as described above, then detached and examined as on day A.

### 4.2. Exposure to EMP

The methodology used in these experiments is based on the RS-15 test of the MIL-461 military electromagnetic compatibility standard, only with much more powerful pulses. It is similar to electroporation techniques [43], and it can simulate exposition to log frequency electromagnetic weapons as explosive magnetic flux compression devices.

All the experiments were conducted in a shielded, semi-anechoic chamber. The attenuation of the chamber was higher than 100 dB for the frequency range of 10 kHz–1 GHz. The electromagnetic pulses were generated with a 600 kV Marx generator connected to the pulse-shaping capacitor feeding a 100 Ω stripline of 50 cm height and 80 cm width. The electric field within the line was controlled by a D-dot sensor connected through an opto-link to an integrating oscilloscope. 

The 12-stage Marx generator was built of 3 nF capacitors charged up to 50 kV by a computer-controlled DC source. Compressed air was used to control the output pulse magnitude. The generator could be triggered electrically by a trigatron at the first stage or could work in a self-triggered mode, delivering up to 10 pulses per second.

The unipolar pulse, shaped by an output capacitor, was similar to MIL-461 RS-105, yet it had a 20 times higher magnitude (reaching 1 MV/m), a slower rise (~4 ns for the highest voltage) and a half-time of ~35 ns. 

**Figure 7:**
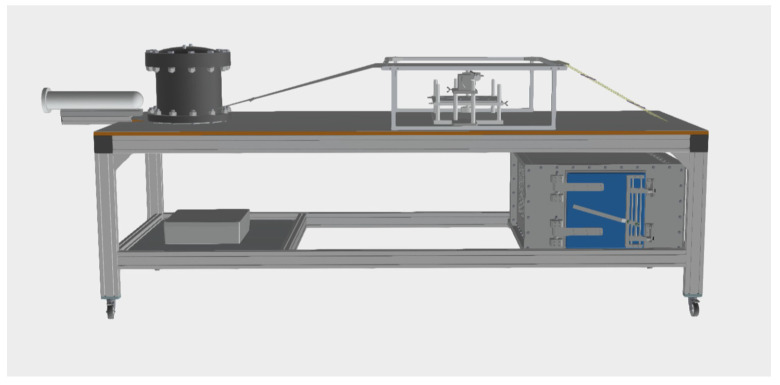
The complete test bed. From left to the right: Marx generator, shaping capacitor, strip line with the working section in the center and terminating resistors on the right-hand side. The screened chamber with the reference cell sample is placed under the stripline below the resistors.

The whole system, i.e., the Marx generator together with the antenna stripline and accessories, was located on the mobile table (3.40 m × 1.15 m × 1.25 m), as shown in Figure 7. In the center of the table, between the antenna and the tabletop, there was an additional smaller table on which the exposed biological samples and the probe were placed. In addition to the elements mentioned above, the system included an additional shielded reference chamber (~80 dB) (located under the top of the mobile table), in which the sham samples were placed—not exposed to the electromagnetic field.

**Figure 8:**
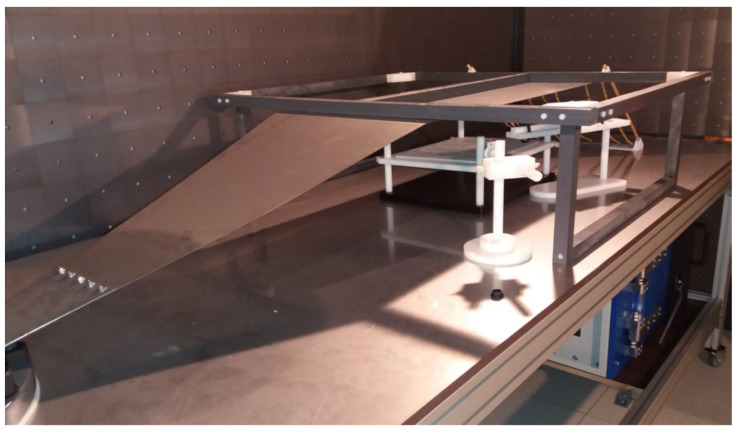
The working part of the stripline.

The working part of the stripline is shown in Figure 8. The stands for the cells and the controlling D-dot probe can be seen in the background, along with the terminating resistor chains.

In the experiments described in this work the cells were exposed to a single pulse with the parameters shown in Table 1.

### 4.3. The Viability and Morphology of the hMSC after Exposure

The viability and morphology of the hMSC were observed and measured at two time points: 2 and 24 h after the exposure. For both the scanning confocal microscope and the SEM observations, three samples were prepared for each group: control, sham, and exposure, from each of the two individual experiments in order to avoid random preparation errors. The hMSC (those exposed and not exposed to the EMP), were stained with fluorescent dyes (LIVE/DEAD™ Viability/Cytotoxicity Kit, Invitrogen™, Waltham, MA, USA) to analyze their viability. Two dyes were used following the manufacturer’s instructions: (1) 0.5 µM calcein A, which stains the cells green with active intracellular esterase and intact membranes, and (2) 4 µM ethidium homodimer 1 (EthD-1), which stains the cells with damaged membranes red. The stained cells were observed under the scanning confocal microscope (LSM 700 Axio Observer.Z1 Zeiss, Wetzlar, Germany). 

The morphology of the hMSC was observed using the SEM (Quanta FEG250, FEI, Hillsboro, OR, USA). The cells (exposed and unexposed) were fixed with paraformaldehyde (PFA 4%, Sigma-Aldrich, Burlington, MA, USA) and glutaraldehyde (GA 0.4%, Sigma-Aldrich, Burlington, MA, USA). Then, the cells were postfixed with osmium tetroxide (Sigma-Aldrich, Burlington, MA, USA) and dehydrated with increasing concentrations of ethanol (POCH, Gliwice, Poland) and acetone (POCH, Gliwice, Poland) as described in our previous paper [44]. The cells were dried using a critical point dryer (Leica EM CPD300, Wetzlar, Germany) and sputtered with 8 nm platinum using a sputter coater (Leica EM ACE200, Wetzlar, Germany). The Everhart–Thornley detector (ETD) working in high vacuum mode was used to observe the cells.

### 4.4. EPR Studies

#### 4.4.1. Preparation of the Samples for EPR Examinations

**Figure 9:**
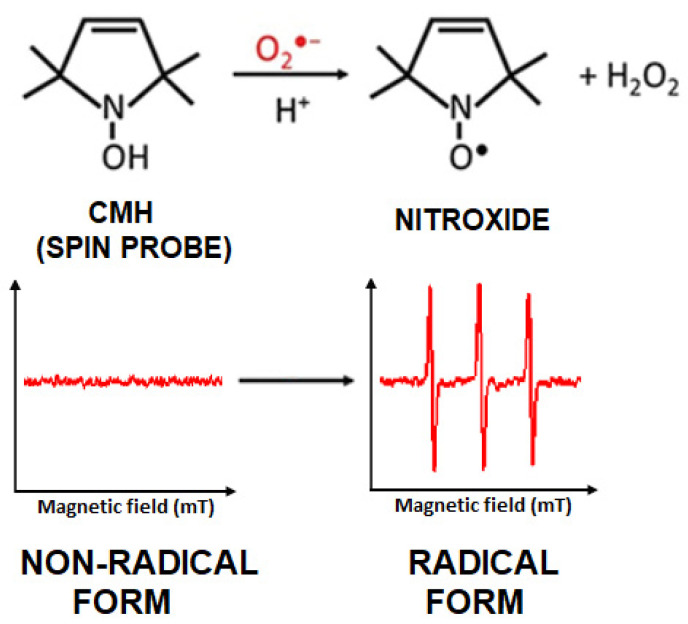
CMH reaction in the presence of O_2_^•−^.

The cell suspensions from the control, sham, and exposed samples were placed in separate tubes on ice to slow down the metabolic processes immediately after harvesting. The cells were centrifuged, and the pellet was suspended in a 3 mM PBS (D-PBS, Dulbecco’s-PBS, Gibco^TM^, Billings, MT, USA) with 25 µM EDTA (Invitrogen^TM^, Carlsbad, CA, USA) to a final density of 2 × 10^6^ cells/mL. The addition of EDTA prevents Fenton’s reactions by chelating metals in the suspensions. The spin trap CMH (1-hydroxy-3-methoxycarbonyl-2,2,5,5-tetramethylpyrrolidine) (Enzo^®^, New York, NY, USA) was added to the cell suspensions to a final concentration of 48.5 mM. The chemical reaction that occurred is shown in Figure 9. The rapid reaction of the CMH probe with O_2_^•−^ radicals that produced a stable CM^•^ nitroxide was quantitatively recorded by the EPR. (It is worth emphasizing that during the exposure of hMSC samples to EMP, other radical forms can be formed, however it is not possible to detect them as the spin trap (CMH) used is selective only to one type of ROS: O_2_^•−^). 

The cell suspensions were vortexed for 2 s and incubated for 2 h. Then they were mixed and transferred to 50 μL capillaries (Blaubrand^®^, Wertheim, Germany). The total volume of the cell suspensions (~4.6 × 10^4^ cells) was 3.14 × 10^−7^ m^3^. The capillaries with cells were stored at 4 °C until measurement.

#### 4.4.2. EPR Measurements

The number of free radicals in the samples, both exposed and not exposed to the EMP, was determined using EPR spectroscopy. The EPR examinations were carried out using an X-band Bruker EMXPlus spectrometer (9.5 GHz) with a magnetic field second modulation frequency of 100 kHz and at 2 °C. The spectrometer was integrated with a temperature controller (Bruker, Reutlingen, Germany). The EPR spectra were registered in a magnetic field sweep range of 5 mT, with a sweep time of 40 s and an attenuation of 23 dB. The standard strong pitch sample (Bruker BioSpin, Billerica, MA, USA) with 1.37 × 10^16^ spins was used to determine the number of free radicals in the samples. The number of free radicals in the cell samples was calculated from the integrated intensity of the free radical signal. The average value of the free radicals and the standard deviation were calculated based on three measurements for each sample group: control, sham, and exposed for each experiment (no.1 and no.2). The Student’s *t*-test was used to check the significance of statistical differences between the groups of samples.

### 4.5. Interaction of a Single High-Energy Electromagnetic Pulse with Biological Material Placed in Cell Culture Flask—A Simulation

The simulations were performed using the Microvawes & RF (Biomedical, Exposure, SAR) package of CST^®^ STUDIO SUITE^®^ 2021.05 software.

**Figure 10:**
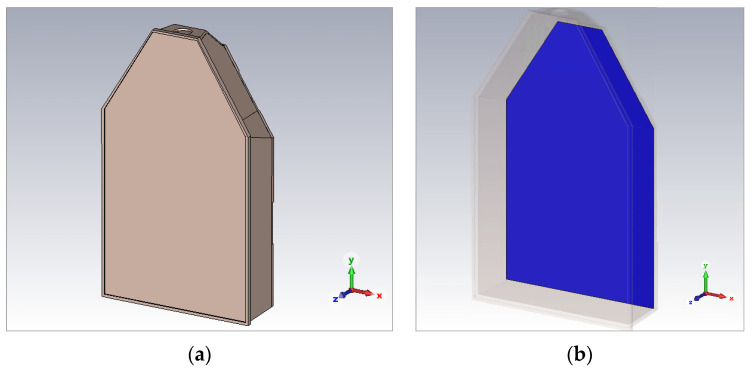
Projection of the T175 culture flask: (**a**) non-transparent, (**b**) transparent—with a visible thin layer of biological material.

The subject of the simulation was a T175 culture flask (Nunc^®^) with a layer of biological material of bone marrow cells with a volume of 10 mL and a layer thickness of 0.588 mm. In the simulation, the specific absorption rate (SAR) distribution (2):

(2) $$SAR=\frac{1}{V}\int_{sample}\frac{\sigma \left(r\right){\left|E\left(r\right)\right|}^{2}}{\rho \left(r\right)}dr$$

(where *σ* is the sample electrical conductivity, E is the root mean square value of the electric field, ρ is the sample density, and *V* is the volume of the sample) was calculated for a flat electromagnetic wave pulse (with an electric field magnitude of ~1 MV/m) falling perpendicularly on the culture flask (Figure 10).

**Figure 11:**
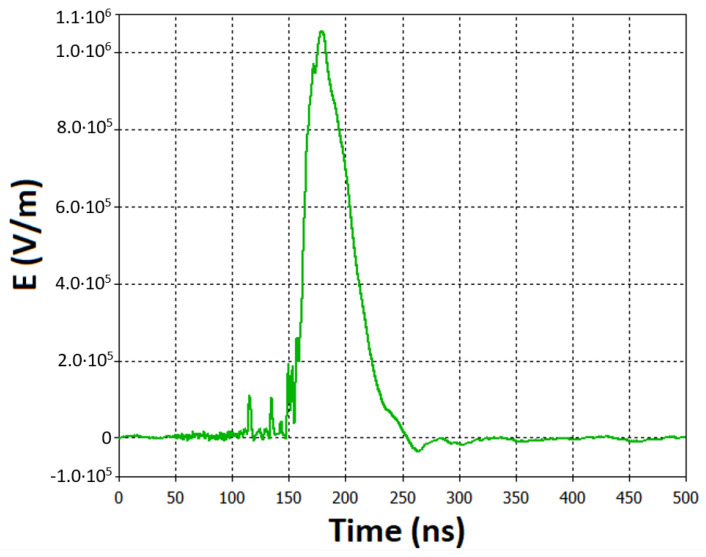
Changes in time of the electrical component of an electromagnetic pulse (data obtained from the Teledyne LeCroy (640 Zi) oscilloscope). Data was used for the calculations in CST^®^ STUDIO SUITE^®^ 2021.05 software.

The values of electric field data obtained during the experiment using the Teledyne LeCroy oscilloscope (640 Zi) (Chestnut Ridge, NY, USA) were used for the calculations made in the CST^®^ STUDIO SUITE^®^ 2021.05 software (Figure 11).

## 5. Conclusions

In this work the effect of single electromagnetic pulse with a magnitude of ~1 MV/m and a duration of ~120 ns on the viability, morphology, and generation of free radicals in hMSC was investigated. Both the cell viability and the EPR measurements showed that the experimental parameters used influenced neither the number of free radicals generated nor a change in the morphology and metabolic activity of hMSC compared to control samples. The usage of the EPR technique to measure free radicals in exposed biological samples was a novelty in this preliminary investigation. In our opinion, the EPR technique is still highly underestimated, and our work could be considered as a trigger which will promote this technique as a precise quantitative method for determining in vitro the number of free radicals in cells after treatment with external physical factors, such as EMP.

## Figures and Tables

**Table 1:** Parameters of the electromagnetic pulse: electric field magnitude, number of pulses, pulse duration, rise time, and half-width time.

Experiment No.	Electric Field Magnitude [kV/m]	Number of Pulses	Pulse Duration [ns]	Rise Time [ns]	Half-Width Time [ns]
1	944	1	117.8	3.7	34
2	1018	1	120	4.1	35

## Data Availability

Data available for a special request of an interested reader (contact with corresponding author).
